# Multi-label auxiliary decision-making model for pharmacotherapy of ischemic stroke based on BR-SA-LightGBM using real-world electronic medical records

**DOI:** 10.3389/fmed.2026.1902875

**Published:** 2026-10-27

**Authors:** Yueqi Chen, Chen Rui, Shuaiyu Mu, Shengfa Zhang, Haoran Liu

**Affiliations:** 1 School of Public Health, Hebei University, Baoding, Hebei, China; 2 School of Economics and Management, Hebei University of Technology, Tianjin, China; 3 Institute of Hospital Management, Cangzhou Central Hospital, Cangzhou, Hebei, China; 4 National Population Health Data Center, Institute of Medical Information, Chinese Academy of Medical Sciences, Beijing, China

**Keywords:** auxiliary decision-making, ischemic stroke, lightgbm, multi-label learning, pharmacotherapy, simulated annealing

## Abstract

**Background:**

Ischemic stroke poses a substantial global health burden owing to its high prevalence, recurrence, disability, and mortality rates. Pharmacotherapy for ischemic stroke is highly heterogeneous across patients, with complex polypharmacy regimens and intricate interdependencies between drug classes. Precise, personalised medication recommendations are therefore indispensable for optimising therapeutic outcomes and improving patient prognosis.

**Methods:**

This study framed pharmacotherapeutic decision-making for ischemic stroke as a multi-label learning task. Using retrospective electronic medical records from 7,335 ischemic stroke patients admitted to a single tertiary hospital (Cangzhou Central Hospital), we developed and evaluated an integrated model, termed BR-SA-LightGBM, which combines Binary Relevance (BR), Simulated Annealing (SA), and the Light Gradient Boosting Machine (LightGBM) to provide personalised recommendations across eight first-line drug categories.

**Results:**

In an independent test cohort (*n* = 2,200), the BR-SA-LightGBM model achieved an accuracy of 0.90, an F1-score of 0.87, and a markedly low Hamming loss of 0.09. It significantly outperformed ML-KNN, Power-Set, and the unoptimized BR-LightGBM model across all evaluation metrics (*P* < 0.05). Across drug classes, age, the number of comorbidities, atrial fibrillation, carotid plaque, and fibrinogen were among the most influential predictors of medication selection.

**Conclusions:**

The BR-SA-LightGBM model established herein enables accurate, personalised, auxiliary decision support for drug prescribing in ischemic stroke. It is intended as an auxiliary decision-support tool that has the potential to reduce medication errors and omissions and may assist clinicians in improving clinical efficiency; it also provides a novel methodological framework for precision medicine in stroke care.

## 1 Introduction

Ischemic stroke represents one of the most prevalent cerebrovascular disorders, characterised by high morbidity, recurrence, disability, and socioeconomic cost, thereby severely threatening public health and quality of life (1). Pharmacotherapy constitutes the cornerstone of acute and secondary preventive management of ischemic stroke. However, substantial inter-individual variability—including age, comorbidities, clinical manifestations, and laboratory profiles—dictates highly personalised medication strategies (2). Clinical practice commonly requires combined prescription of antiplatelet, anticoagulant, defibrinogenating, vasodilatory, neuroprotective, volume-expanding, statin, and anti-infective agents, selected and adjusted according to individual patient status (3). Traditionally, prescribing decisions rely heavily on clinician experience and guideline recommendations. When confronted with high-dimensional clinical data and diverse therapeutic options, however, clinicians face elevated risks of suboptimal drug selection, incomplete regimens, or preventable adverse events, all of which compromise therapeutic efficacy and patient safety (4).

Against this background, the expanding availability of electronic medical record (EMR) data and advances in machine learning have catalysed the development of data-driven decision-support systems for medication prescription (5). Multi-label learning is particularly well-suited to this clinical scenario, as it addresses classification problems in which a single patient may require multiple drug labels. This framework aligns naturally with the polypharmacy nature of ischemic stroke management and offers distinct advantages for personalised medication recommendations (6). Nevertheless, existing applications of multi-label learning in stroke pharmacotherapy remain limited: many models rely on simplistic label-transformation strategies that disregard inherent correlations between drug classes (7, 8); base classifiers are often poorly tuned, thereby limiting predictive performance; and many systems cover an insufficient range of medications, limiting real-world utility (9).

LightGBM is a highly efficient gradient-boosting decision tree (GBDT) algorithm known for computational speed, low memory usage, and strong resistance to overfitting, making it widely applicable in medical data mining (10, 11). Simulated Annealing (SA) is a robust global optimisation algorithm that mitigates premature convergence to local optima during parameter tuning. Binary Relevance (BR) is a classic, computationally efficient, and scalable multi-label transformation strategy that decomposes complex multi-label tasks into independent binary subproblems, rendering it suitable for high-dimensional clinical datasets (12). By synergizing these three methodologies, we constructed the BR-SA-LightGBM model to enhance the accuracy, reliability, and personalization of drug recommendations for ischemic stroke. Article types.

This study aimed to develop and validate an accurate, personalised multi-label auxiliary decision-support model for pharmacotherapy in patients with ischemic stroke. Using a multi-label learning framework, we classified therapeutic agents for ischemic stroke and integrated the BR method with SA-optimised LightGBM to enable data-driven medication recommendations. We further identified key determinants of drug selection across categories to provide interpretable, clinically actionable evidence for rational prescribing.

We developed a multi-label auxiliary decision-making model tailored to the complexity and polypharmacy characteristics of ischemic stroke pharmacotherapy. Following systematic feature selection and the construction of a comprehensive clinical indicator system, we integrated BR with the SA-tuned LightGBM algorithm to build a predictive decision-support tool. The model generates individualised medication recommendations and quantifies feature–drug associations to support evidence-based clinical decision-making.

The key innovations of this study are as follows: (1) A BR-RF (Binary Relevance–Random Forest) feature selection pipeline was implemented to reduce dimensionality while preserving category-specific key features, minimising information loss. (2) Simulated Annealing was coupled with LightGBM to optimise base classifier parameters for each binary subproblem, substantially boosting overall model performance. (3) Using large-scale real-world EMR data, the model covers eight major drug classes used in ischemic stroke, enabling comprehensive and personalised medication recommendations while uncovering clinically meaningful relationships between patient characteristics and drug utilisation.

## 2 Materials and methods

### 2.1 Data source

This study employed a retrospective cohort design. Data were obtained from the electronic medical record (EMR) database of the Department of Neurology of a tertiary hospital in Cangzhou, Hebei Province. A total of 22,508 inpatients admitted to the Department of Neurology between January 2024 and January 2025 were initially enrolled, including 9,132 patients diagnosed with ischemic stroke and 13,376 patients without ischemic stroke. After applying strict inclusion and exclusion criteria, 7,335 eligible patients were retained for analysis. The dataset was partitioned into a training set (*n* = 5,135) and a test set (*n* = 2,200) at a 7:3 ratio using stratified random sampling to preserve class distributions. Details of the inclusion and exclusion criteria are shown in Figure 1.

**Figure 1:**
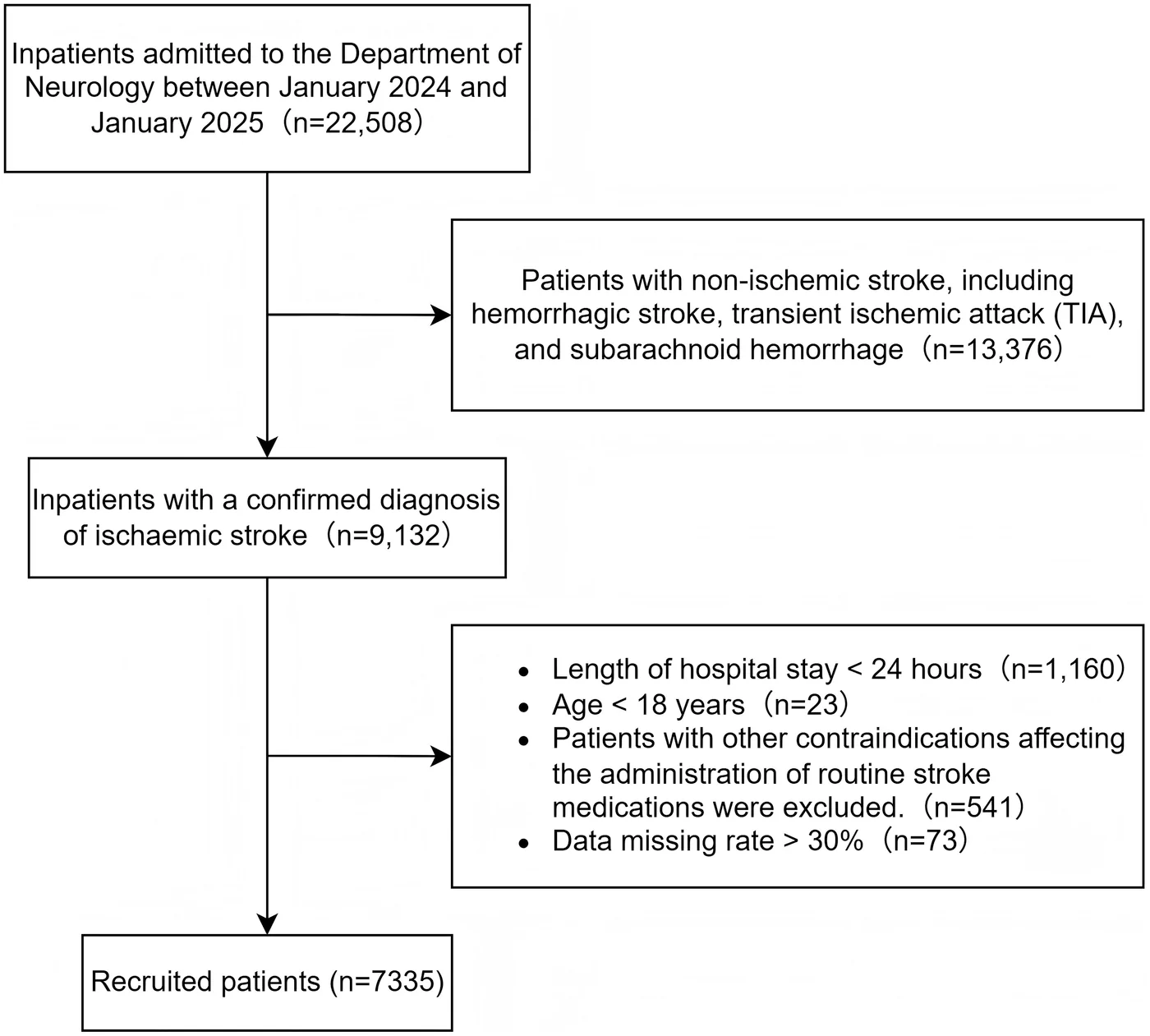
Flowchart of the originally reported patient selection. The source report screened 22,508 neurology inpatients, identified 9,132 with ischemic stroke, and retained 7,335 after the listed exclusions.

### 2.2 Data preprocessing

#### 2.2.1 Initial indicator system design

Therapeutic medications for patients with ischemic stroke are selected based on demographic characteristics, clinical symptoms, physical examinations, and physician prescriptions. Extracting individual patient conditions from electronic medical records (EMRs) to establish an indicator system is a critical foundation for constructing an auxiliary decision-making model for pharmacotherapy in ischemic stroke.

Demographic characteristics mainly include gender, age, and medical history. According to previous studies, age and complications—such as hypertension, heart disease, diabetes, hyperlipidemia, pulmonary infection, bronchitis, and upper respiratory tract infection—exert substantial impacts on medication selection (13). Notably, the number of complications influences drug choice in ischaemic stroke patients (14). Ischemic stroke remains a major clinical challenge, and most patients present with multiple comorbidities, resulting in highly complex medication regimens. Therefore, prior medical history must be incorporated when recommending drugs, and strict control of complications is essential to optimise clinical outcomes.

Admission symptoms provide essential guidance for pharmacotherapy in ischemic stroke. Clinical manifestations, including dizziness, headache, and limb numbness, are strongly associated with medication decisions (15). Physicians typically rely on these symptoms to assess initial medication requirements; thus, relevant clinical features play a key role in drug recommendations. Likewise, standard stroke scales, as objective measures of neurological status, are strongly associated with medication decisions and also serve as key clinical indicators (16). The National Institutes of Health Stroke Scale (NIHSS), the modified Rankin Scale (mRS), the Glasgow Coma Scale (GCS), and the Activities of Daily Living (ADL) score were all incorporated into the initial indicator system; among them, NIHSS, mRS, and ADL scores entered the final feature set and contributed to the model's feature weights, whereas GCS was excluded before modelling because of missing data.

Laboratory and imaging indicators also provide critical evidence to guide medication. In studies of stroke-related biomarkers, total bilirubin, lymphocyte percentage, creatinine, platelets, and haemoglobin serve as important reference factors for treatment planning (17). Imaging features—such as plaque, softened foci, and leukoaraiosis—are highly informative for selecting therapeutic agents (18). Imaging findings precisely identify the location and aetiology of ischemic stroke, supporting targeted pharmaceutical care for different stroke subtypes (19). Clinical experience indicates that stent implantation affects subsequent medication regimens, as patients who undergo stenting usually require specific drugs for post-operative recovery. Accordingly, stent status was included in the indicator system.

In this study, the initial indicator system for the auxiliary decision-making model of pharmacotherapy in patients with ischemic stroke was constructed from 8 dimensions: demographic data, imaging characteristics, admission symptoms, scale scores, admission physical signs, primary treatment information, laboratory indicators, and clinical history (Table 1).

**Table 1:** Initial index system of the drug assistant decision model for ischemic stroke.

Database table	Variables
Demographic data	Gender, age
Imaging features	Intracranial infection, malacic foci, leukoaraiosis, cerebral artery stenosis, cerebral atrophy, intracranial calcification, cerebral atherosclerosis, intracranial plaque, hypodense lesions, cerebral vascular occlusion, cerebral effusion
Admission symptoms	History of antibiotic allergy, dizziness, and vertigo, hemiplegia and aphasia, limb numbness, cognitive impairment, convulsions, and increased muscle tone
Scale scores	ADL score (activities of daily living), admission NIHSS score (National Institutes of Health Stroke Scale), admission mRS score (modified rankin scale), GCS score (glasgow coma scale)
Admission physical signs	Systolic blood pressure, diastolic blood pressure, body temperature, pulse rate, BMI (body mass index)
Main treatment information	Stent implantation (Yes/No)
Laboratory indicators	Low-density lipoprotein cholesterol (LDL-C), Thrombin time (TT), Homocysteine, Uric acid, Total bilirubin, White blood cell count (WBC), Red blood cell count (RBC), Creatine kinase (CK), Platelet count, Blood glucose, Haemoglobin, Fibrinogen, Lymphocyte percentage, Mean corpuscular volume (MCV), Triglycerides, Direct bilirubin, High-density lipoprotein cholesterol (HDL-C), Absolute lymphocyte count, Mean corpuscular haemoglobin concentration (MCHC)
Clinical history	Hypertension, Coronary heart disease (CHD), Diabetes mellitus, Transient ischemic attack (TIA), Peripheral arterial disease (PAD), Postoperative sequelae, Atrial fibrillation (AF), Hyperlipidemia, Respiratory tract infection, Epilepsy, Liver dysfunction, Number of complications, History of trauma

#### 2.2.2 Missing value and outlier handling

Because EMR data commonly contain noise, coding errors, and missing values, a systematic data-quality procedure was applied before modelling, comprising missing-value handling and outlier detection and review. First, variables with a missing rate exceeding 90% were excluded, and records with more than 70% missing variables were omitted for each patient. For the remaining missing values, normally distributed continuous variables were imputed using the mean; skewed continuous variables were imputed using the median; and discrete variables were imputed using the k-nearest neighbour (k-NN) algorithm based on Euclidean distance. Next, outliers were detected using two complementary approaches: (1) statistical detection using the 3*σ* criterion, where values outside the range *μ* ± 3*σ* (μ = mean, *σ* = standard deviation) were flagged as outliers; and (2) clustering-based outlier detection. Finally, all identified outliers were reviewed and corrected by clinical experts to ensure biological plausibility.

#### 2.2.3 Data standardisation

Continuous variables were min–max normalised to the range [0, 1] to eliminate scale effects:

$$X_{\mathrm{norm}}=\frac{X-X_{\min}}{X_{\max}-X_{\min}}$$

where $X_{\mathrm{norm}}$ is the normalised value, *X* is the original variable value, and $X_{\max}$, $X_{\min}$ are the maximum and minimum values of the variable, respectively.

#### 2.2.4 Categorical feature encoding

Categorical variables were transformed using one-hot encoding, employing a method that converts each discrete feature with ncategories into nbinary features and generates a sparse matrix suitable for machine learning.

#### 2.2.5 Label construction

According to drug classification standards, an 8-dimensional binary label vector was constructed to mark whether patients used specific drug classes. The data label set was generated by sorting out the medication information of patients' doctors' orders. Referring to the Chinese Guidelines for the Diagnosis and Treatment of Acute Ischemic Stroke 2023 (20), therapeutic drugs were divided into 8 categories: antiplatelet, anticoagulant, defibrase, vasodilator, neuroprotective, volume expansion, statin, and anti-infective drugs (21), involving a total of 34 main therapeutic drugs (Table 2).

**Table 2:** Drug classification table based on ischemic stroke treatment guidelines.

Category	Drug names
Antiplatelet agents	Aspirin, Ozagrel, Clopidogrel, urinary kallidinogenase
Volume expanders	Dextran, Hydroxyethyl starch
Statins	Simvastatin, Pitavastatin, Atorvastatin, Rosuvastatin
Anticoagulants	Argatroban, Batroxobin, Dabigatran, Warfarin
Fibrinolytics	Fibrinolysin, Lumbrokinase
Vasodilators	Betahistine mesylate, Nicergoline, Nimodipine, Alprostadil, Difenidol hydrochloride, Flunarizine
Neuroprotective agents	Butylphthalide, Oxiracetam, Cytophosphocholine, Piracetam, Compound aminobutyric acid, Annao pills
Anti-infective agents	Acyclovir, Aztreonam, Caspofungin acetate, Flucloxacillin sodium, Meropenem, Minocycline

According to the above drug classification, the 8 drug categories were sorted and calculated. The label counts of each category of ischemic stroke therapeutic drugs are shown in Table 3.

**Table 3:** Statistical information dataset of therapeutic drugs for ischemic stroke.

Category	Label count
Antiplatelet agents	7,251
Volume expanders	1,256
Statins	887
Anticoagulants	369
Fibrinolytics	596
Vasodilators	5,397
Neuroprotective agents	7,153
Anti-infective agents	843

### 2.3 Model architecture

#### 2.3.1 Binary relevance (BR)

The core idea of the BR method is to decompose the multi-label classification problem into a set of independent binary classification subproblems (22). Let the label set be *L* = l1, *l*_2_, ., *l_q_* (*q* = 8 in this study, corresponding to 8 drug categories). For each label *l_j_* (*j* = 1, 2, .., 8), a binary classification subproblem was constructed to determine whether patients require medications from the *j*-th therapeutic class was encoded as a binary indicator. The original dataset was partitioned into eight binary classification tasks based on these labels, and dedicated base classifiers were trained for each task. Finally, the outputs of all base classifiers were aggregated to form the multi-label prediction result. The BR model structure is shown in Figure 2.

**Figure 2:**
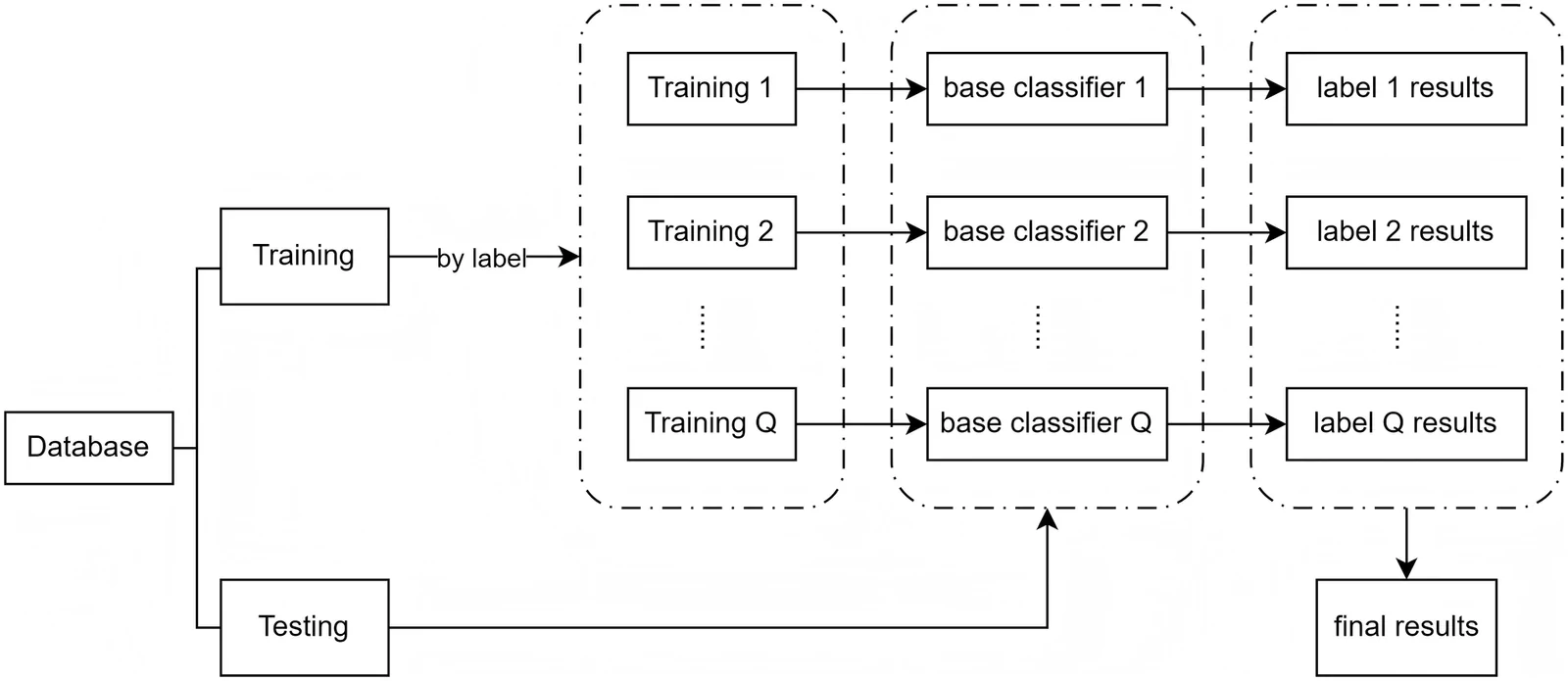
Binary relevance (BR) architecture. Eight binary classifiers predict recorded use of the eight drug categories, and their outputs are combined into a multi-label vector.

#### 2.3.2 LightGBM algorithm

LightGBM is an advanced GBDT implementation that employs Gradient-based One-Side Sampling (GOSS) and Exclusive Feature Bundling (EFB) to accelerate training and improve scalability for high-dimensional data (23, 24). GOSS retains samples with large gradients and randomly samples those with small gradients to reduce computation. EFB bundles mutually exclusive sparse features to reduce effective dimensionality.

#### 2.3.3 Simulated annealing (SA)

SA is a heuristic global optimisation algorithm inspired by the thermal annealing process in metallurgy, which effectively escapes local optima (25). The procedure is as follows: (1) Initialization: Define an initial parameter set *x*_0_, initial temperature *T*_0_, stopping temperature Tend, and cooling rate d. (2) Iterative search: generate a new solution *x*_1_ from the neighbourhood of the current solution at the current temperature, calculate the objective function value *f* (*x*_1_). If *f* (*x*_1_) < *f* (*x*_0_), accept the new solution; otherwise, determine acceptance based on the Metropolis criterion. (3) Cooling: *T_i_* _+_ _1_ = *d* (*T_i_*). If the temperature is lower than the termination temperature, terminate the iteration and output the optimal solution. As a general optimization algorithm, this algorithm effectively mitigates the risk of convergence to local minima and excessive sensitivity to initial values and has demonstrated robust performance across diverse application domains.

#### 2.3.4 BR-SA-LightGBM model flow

The overall model flow is as follows:

Input: Original dataset *U*, where the label set is *L* = {*l*1, *l*2, .., *l*q}.
Step 1: The original dataset is processed by feature selection to obtain dataset *T*, which is divided into a training set *T_m_* and a test set *T_k_* at a ratio of 7:3. The feature set of the training set is denoted as *T_md_*, and the label set is denoted as *T_ml_*.
Step 2: For each label *l_j_* (*j* = 1, 2, .., *q*) in the label set of the training set, combine it with the training data feature set *T_md_* to form a training dataset *T_mdj_*. In this study, *j* = 1 corresponds to the first label: antiplatelet therapeutic drugs.
Step 3: Construct a base classifier *p_j_* for whether to use this category of drugs based on *T_mdj_*, train the base classifier *p_j_* using LightGBM, and optimize parameters using the simulated annealing algorithm.
Step 4: Repeat steps 2–3 for the *j*-th (*j* = 1,2,3,.., *q*) label to complete the training of all base classifiers. In this study, SA-optimised LightGBM models were sequentially constructed for anticoagulant, defibrase, vasodilator, neuroprotective, volume expansion, statin, and anti-infective drugs according to steps 1–2. Then, *q* labels were traversed and trained to form the final multi-label learning model.
Step 5: For a sample *k_i_* with unknown labels in the test set *T_k_*, predict *k_i_*. If *p_j_* (*k_i_*) = 1, the ischemic stroke patient needs to use the *j*-th category of drugs for treatment; otherwise, *p_j_* (*k_i_*) = 0, indicating that the patient does not use the *j*-th category of drugs. The associated label set *L^'^_K_* is predicted by the *q* pre-trained base classifiers.
Output: The predicted associated label set *L^'^_K_* of the sample *k_i_* with unknown labels in the test set and the multi-label learning model evaluation indicators.

In this study, patient admission and diagnostic data were utilized to train the model and explore patterns of multi-label medication utilization. The test set was employed to evaluate the model's predictive performance on unseen samples and to assess its stability and generalizability (Figure 3).

**Figure 3:**
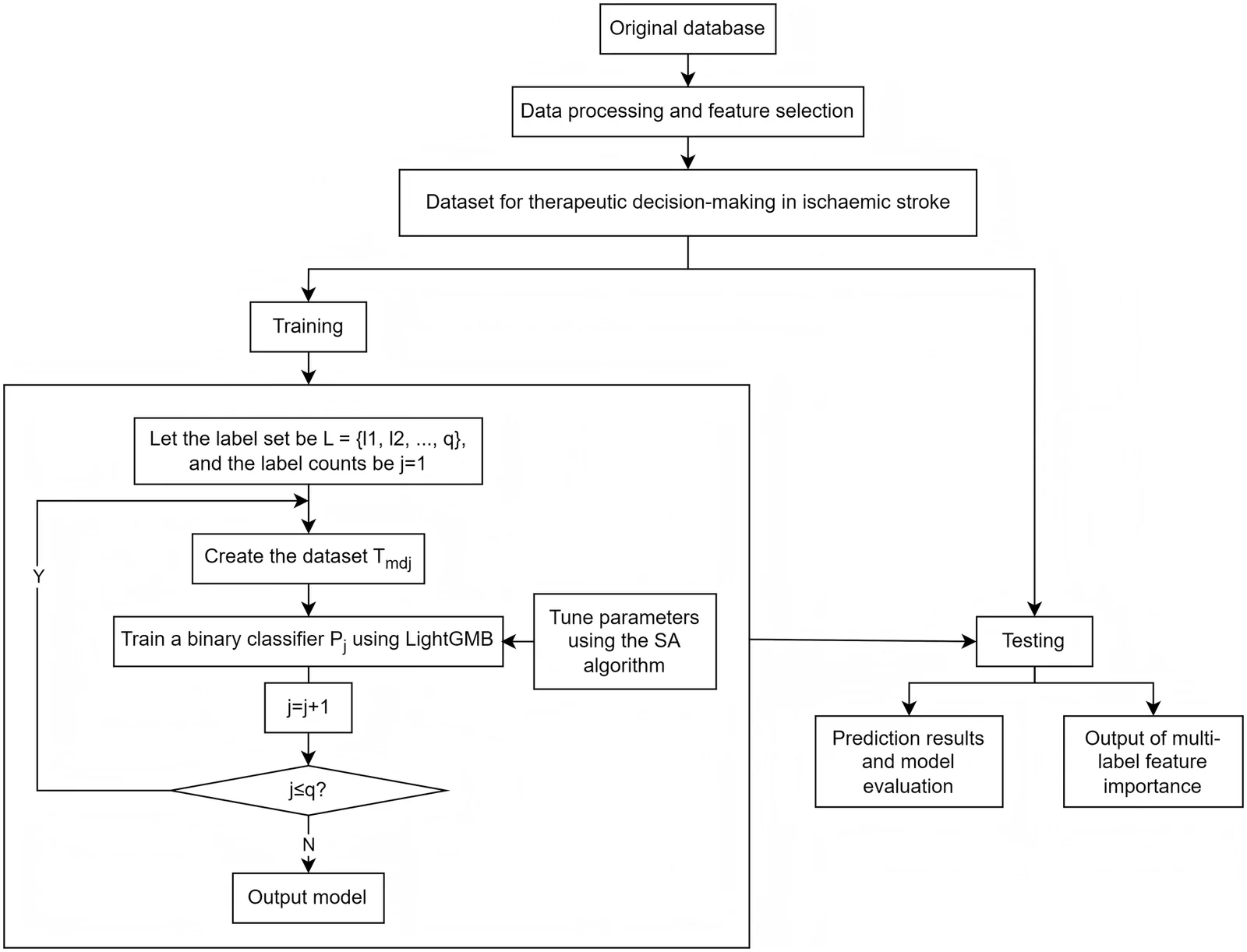
Logical diagram of drug treatment decision support model for ischemic stroke based on BRSA-LightGBM. LightGBM is used for each binary drug label and SA searches classifier hyperparameters.

### 2.4 Feature selection and descriptive statistics

Based on the characteristics of the initial indicator system for ischemic stroke drug therapy auxiliary decision-making, the BR-RF algorithm was used for feature selection to identify key predictors associated with drug prescription patterns in ischemic stroke patients, enhance algorithmic computational efficiency, eliminate irrelevant variables, and mitigate the complexity of model training. Given that the BR framework comprises multiple binary classifiers, the integrity of each label of the entire algorithm should be ensured during feature selection. This study screened all features of the 8 label classifications one by one, combined with the BR method, used random forest to screen features, and took the union of the selected features into the model. The steps of integrated multi-label feature selection are shown in Figure 4.

**Figure 4:**
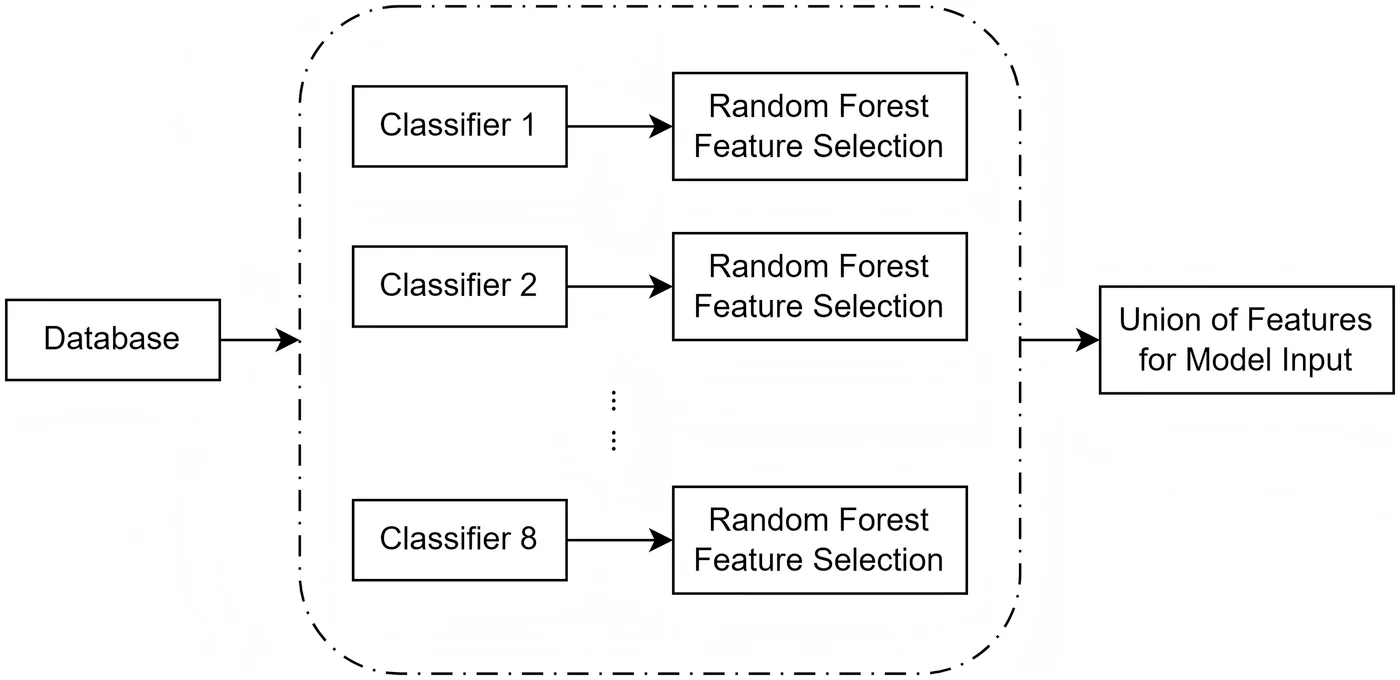
Feature selection structure map based on BR-RF. A random forest ranks candidate variables separately for each binary drug label; the ten highest-ranked variables per label are combined by union.

The dataset was partitioned into eight subsets based on drug labels. To prevent information leakage, all feature selection was performed exclusively on the training set after the 7:3 data split, and the test set was never accessed during feature selection. Random forest was employed to perform feature selection on each subset, and the top 10 features with the highest feature importance scores were retained for each of the eight binary classifiers. The union of these selected features comprised 38 variables that entered the final models. The final indicator system of the ischemic stroke drug therapy auxiliary decision-making after algorithm screening is shown in Table 4.

**Table 4:** Variable information of the final index system of the drug therapy assistant decision model for ischemic stroke.

Category	Variables	Variable description	Variable type and settings
Demographic data	Age	Patient's age	Numeric
	Gender	Gender	1, Male; 2, Female
Signs on admission	Temperature	The patient's body temperature on admission	Numeric
	BMI	The patient's BMI is calculated from their height and weight according to national standards	Numeric
	Systolic blood pressure	The patient's systolic blood pressure measured on admission	Numeric
	Pulse rate	The patient's pulse rate per minute measured on admission	Numeric
	Diastolic blood pressure	The patient's diastolic blood pressure measured on admission	Numeric
Imaging features	Cerebrospinal fluid	Presence of effusion on brain imaging	0 = Absent; 1 = Present
	Infection	Presence of infection on brain imaging	0 = Absent; 1 = Present
	Cerebrovascular stenosis	Presence of stenosis in cerebral blood vessels	0 = Absent; 1 = Present
	Atherosclerosis	Presence of atherosclerosis in cerebral blood vessels	0 = Absent; 1 = Present
	Plaques	Presence of plaques on brain imaging	0 = Absent; 1 = Present
	Calcification	Presence of calcification in the brain	0 = Absent; 1 = Present
	Low-density lesion	Presence of hypodense lesions on brain imaging	0 = Absent; 1 = Present
	Leukoaraiosis	Presence of leukoaraiosis	0 = Absent; 1 = Present
	Softening lesion	Presence of malacic foci on brain imaging	0 = Absent; 1 = Present
	Obstruction	Presence of occlusion in cerebral blood vessels	0 = Absent; 1 = Present
Lifestyle	Smoking	History of smoking	0 = No; 1 = Yes
Clinical history	Number of complications	Total number of complications in the patient	Numeric
	Atrial fibrillation	Presence of atrial fibrillation	0 = Absent; 1 = Present
	Hypertension	Presence of hypertension	0 = Absent; 1 = Present
	Peripheral arterial disease	Presence of arteriosclerosis obliterans, arteriovenous thrombosis, or related conditions	0 = Absent; 1 = Present
	Coronary heart disease	Presence of coronary heart disease, including myocardial ischemia, angina pectoris, myocardial infarction, and heart failure	0 = Absent; 1 = Present
	Diabetes mellitus	Presence of diabetes mellitus, including type 1 and type 2 diabetes	0 = Absent; 1 = Present
	Hyperlipidemia	Presence of hyperlipidemia	0 = Absent; 1 = Present
	Respiratory tract infection	Presence of rhinitis, pharyngitis, bronchitis, or related symptoms	0 = Absent; 1 = Present
Scale scores	ADL score	Score on the activities of daily living (ADL) scale	Numeric
	NIHSS score	Score on the national institutes of health stroke scale (NIHSS)	Numeric
	mRS score	Score on the modified rankin scale (mRS)	Numeric
Laboratory indicators	Fibrinogen	Unit: g/L; reference range: 1.8–4	0 = Low; 1 = Normal; 2 = High
	Lymphocyte percentage	Unit:%; reference range: 18–40	0 = Low; 1 = Normal; 2 = High
	Homocysteine	Unit: μmol/L; reference range: 4–15.4	0 = Low; 1 = Normal; 2 = High
	Mean corpuscular volume (MCV)	Unit: fL; reference range: 82–95	0 = Low; 1 = Normal; 2 = High
	Triglycerides	Unit: mmol/L; reference range: 0–1.7	0 = Low; 1 = Normal; 2 = High
	Blood glucose	Unit: mmol/L; reference range: 3.9–6.1	0 = Low; 1 = Normal; 2 = High
	Low-density lipoprotein cholesterol (LDL-C)	Unit: mmol/L; reference range: 0–3.12	0 = Low; 1 = Normal; 2 = High
	Absolute lymphocyte count	Unit: × 10^9^/L; reference range: 1–3.3	0 = Low; 1 = Normal; 2 = High
	Direct bilirubin	Unit: μmol/L; reference range: 0–6.8	0 = Low; 1 = Normal; 2 = High
	Mean corpuscular haemoglobin concentration (MCHC)	Unit: g/L; reference range: 320–360	0 = Low; 1 = Normal; 2 = High
	High-density lipoprotein cholesterol (HDL-C)	Unit: mmol/L; reference range: 0.8–1.5	0 = Low; 1 = Normal; 2 = High

Integrating the results of the BR-RF feature selection, descriptive statistics were conducted on the qualitative indicators within the dataset. The data description of qualitative indicators mainly includes the type, setting, quantity, and percentage of each variable. The descriptive information of qualitative variables in the final indicator system of the ischemic stroke drug therapy auxiliary decision-making model is shown in Table 5.

**Table 5:** Statistical description of qualitative variables in the final index system of the drug therapy decision support model for ischemic stroke.

Category	Variable	Variable type and coding	*n*	Proportion
Clinical history	Atrial fibrillation	0 = Absent	7,149	0.9746
		1 = Present	186	0.0254
	Hypertension	0 = Absent	2,039	0.278
		1 = Present	5,296	0.722
	Peripheral arterial disease	0 = Absent	4,418	0.6023
		1 = Present	2,917	0.3977
	Respiratory tract infection	0 = Absent	6,302	0.8592
		1 = Present	1,033	0.1408
	Coronary heart disease	0 = Absent	5,830	0.7948
		1 = Present	1,505	0.2052
	Diabetes mellitus	0 = Absent	4,865	0.6633
		1 = Present	2,470	0.3367
	Hyperlipidaemia	0 = Absent	6,210	0.8466
		1 = Present	1,125	0.1534
Demographic data	Sex	1 = Male	4,696	0.6402
		2 = Female	2,639	0.3598
Lifestyle	Smoking	0 = No	5,439	0.7415
		1 = Yes	1,896	0.2585
Laboratory indicators	Fibrinogen	0 = Low	54	0.0074
		1 = Normal	6,348	0.8654
		2 = High	933	0.1272
	Lymphocyte percentage	0 = Low	1,164	0.1587
		1 = Normal	5,748	0.7836
		2 = High	423	0.0577
	Homocysteine	0 = Low	1	0.0001
		1 = Normal	3,604	0.4913
		2 = High	3,730	0.5086
	Mean corpuscular volume (MCV)	0 = Low	136	0.0185
		1 = Normal	5,032	0.6861
		2 = High	2,167	0.2954
	Triglycerides	0 = Low	5,503	0.7502
		1 = Normal	1,832	0.2498
		2 = High	0	0
	Blood glucose	0 = Low	51	0.007
		1 = Normal	5,576	0.7602
		2 = High	1,708	0.2328
	Low-density lipoprotein cholesterol (LDL-C)	0 = Low	0	0
		1 = Normal	5,431	0.7404
		2 = High	1,904	0.2596
	Absolute lymphocyte count	0 = Low	580	0.0791
		1 = Normal	6,662	0.9082
		2 = High	93	0.0127
	Direct bilirubin	0 = Low	0	0
		1 = Normal	5,583	0.7611
		2 = High	1,752	0.2389
	Mean corpuscular haemoglobin concentration (MCHC)	0 = Low	1,954	0.2664
		1 = Normal	5,380	0.7335
		2 = High	1	0.0001
	High-density lipoprotein cholesterol (HDL-C)	0 = Low	758	0.1033
		1 = Normal	6,140	0.8371
		2 = High	437	0.0596
Imaging features	Cerebrospinal fluid	0 = Absent	6,618	0.9022
		1 = Present	717	0.0978
	Infection	0 = Absent	6,982	0.9519
		1 = Present	353	0.0481
	Cerebrovascular stenosis	0 = Absent	2,416	0.3294
		1 = Present	4,919	0.6706
	Atherosclerosis	0 = Absent	2,512	0.3425
		1 = Present	4,823	0.6575
	Plaques	0 = Absent	2,963	0.404
		1 = Present	4,372	0.596
	Calcification	0 = Absent	4,553	0.6207
		1 = Present	2,782	0.3793
	Low-density lesion	0 = Absent	2,349	0.3202
		1 = Present	4,986	0.6798
	Leukoaraiosis	0 = Absent	4,168	0.5682
		1 = Present	3,167	0.4318
	Softening lesion	0 = Absent	3,680	0.5017
		1 = Present	3,655	0.4983
	Obstruction	0 = Absent	5,447	0.7426
		1 = Present	1,888	0.2574

The quantitative indicators of the dataset were mainly described by the mean and standard deviation of variables. The descriptive information of quantitative variables in the final indicator system of the ischemic stroke drug therapy auxiliary decision-making model is shown in Table 6.

**Table 6:** Statistical description of quantitative variables in the final index system of the adjuvant decision-making model for drug treatment of ischemic stroke.

Category	Variable	Mean	SD
Clinical history	Number of complications	2.69	1.46
Demographic data	Age	62.91	11.00
Admission physical signs	Body temperature	36.49	0.23
	BMI	23.52	3.93
	Pulse rate	73.28	8.39
	Systolic blood pressure	138.31	21.60
	Diastolic blood pressure	84.57	10.61
Scale scores	ADL score	76.33	7.85

### 2.5 Model training and hyperparameter optimisation

This experiment was implemented using the Python language, and the BR-SA-LightGBM model was used for multi-label prediction of medication in ischemic stroke patients. Given that the BR method divides positive and negative sample datasets for each label, it leads to data imbalance. When applying LightGBM as the base classifier, the parameter scale_pos_weight was set to 1 to balance the label data. Since each classifier targets a different dataset, to further improve model accuracy, the simulated annealing algorithm was used to adjust the important parameters of LightGBM. The main parameters to be adjusted are shown in Table 7.

**Table 7:** LightGBM main parameters description table.

Parameter name	Description	Value range
bagging_fraction	Subsample ratio of training instances per iteration	(0,1)
boosting_type	Boosting type	Dart, GBDT, RF
feature_fraction	Subsample ratio of features per iteration	(0,1)
learning_rate	Learning rate	(0.01,0.5)
max_depth	Maximum tree depth	(2,20)
num_leaves	Maximum number of leaves per tree	(10,200)

The parameters of the simulated annealing algorithm were set as follows: initial temperature = 100, termination iteration temperature = 5, attenuation function = 0.95. Then, the parameters of each base classifier were optimized sequentially, and the parameter values of each base classifier are shown in Table 8.

**Table 8:** Each classifier LightGBM parameter values.

Parameter Name	Antiplatelet agents	Volume expanders	Statins	Anticoagulants	Fibrinolytics	Vasodilators	Neuroprotective agents	Anti-infective agents
bagging_fraction	0.9710	0.9376	0.7522	0.8764	0.7001	0.5995	0.8166	0.8725
bagging_freq	4	5	10	9	17	11	3	17
boosting_type	gbdt	dart	dart	gbdt	dart	rf	gbdt	gbdt
feature_fraction	0.8248	0.8412	0.7429	0.7943	0.8222	0.8485	0.8493	0.8886
learning_rate	0.0203	0.3467	0.0117	0.0071	0.0131	0.0642	0.1344	0.0161
max_depth	7	10	5	6	3	7	10	7
num_leaves	111	124	55	47	121	77	85	28

### 2.6 Model evaluation metrics

Four multi-label evaluation indicators were used to evaluate the overall effect of the multi-label classification model and compare it with four other mainstream multi-label classification methods: Hamming Loss, Accuracy, Recall, and F1-score.

In addition to the four overall metrics, per-class sensitivity, specificity, precision, recall, and F1-score were calculated for each drug category, together with a category-specific confusion matrix. Because the prevalence of the eight drug classes differed markedly, an operating threshold was selected independently for each classifier using three-fold out-of-fold predictions on the training set. The per-class results are reported at the Youden-optimal threshold, which maximises the Youden index J and balances sensitivity and specificity (Tables 9, 10); the overall performance of the proposed model is reported at the per-class F1-optimal thresholds, which represent the recommended deployment operating point (Table 11). The fixed threshold of 0.5 is additionally reported for reference, and the test set was never used for threshold selection.

**Table 9:** Per-class performance of the eight binary classifiers on the independent test set at the Youden-optimal operating threshold.

Drug class	*n* (positive)	AUC	Threshold	Sensitivity	Specificity	Precision	F1
Antiplatelet agents	2,175	0.962	0.998	0.787	0.960	0.998	0.881
Anticoagulants	111	0.917	0.058	0.622	0.944	0.371	0.465
Defibrinogenating agents	170	0.927	0.334	0.847	0.936	0.527	0.650
Vasodilators	1,619	0.672	0.694	0.780	0.470	0.804	0.792
Neuroprotective agents	2,145	0.796	0.051	0.492	0.836	0.992	0.658
Statins	266	0.903	0.71	0.741	0.900	0.505	0.601
Volume expanders	377	0.660	0.412	0.780	0.440	0.224	0.348
Anti-infective agents	238	0.921	0.119	0.634	0.893	0.418	0.504

AUC is the held-out area under the receiver-operating-characteristic curve; sensitivity, specificity, precision, and F1-score correspond to the selected threshold.

**Table 10:** Category-specific confusion matrices on the independent test set at the Youden-optimal thresholds.

Drug class	TP	FP	TN	FN
Antiplatelet agents	1,712	1	24	463
Anticoagulants	69	117	1,972	42
Defibrinogenating agents	144	129	1,901	26
Vasodilators	1,263	308	273	356
Neuroprotective agents	1,055	9	46	1,090
Statins	197	193	1,741	69
Volume expanders	294	1,021	802	83
Anti-infective agents	151	210	1,752	87

TP, true positive; FP, false positive; TN, true negative; FN, false negative.

**Table 11:** Comparison table of evaluation indicators for each model.

Algorithm	Accuracy	Recall	F1-score	Hamming loss
ML-KNN	0.84	0.79	0.67	0.15
Power-Set	0.64	0.44	0.54	0.35
MLARAM	0.85	0.77	0.75	0.14
BRKNN	0.85	0.78	0.74	0.14
BR-LightGBM	0.88	0.87	0.86	0.11
BR-SA-LightGBM	0.90	0.91	0.87	0.09

To assess the stability of the performance estimates beyond the single 7:3 random split, a stratified five-fold cross-validation was performed over the entire cohort. The per-fold area under the receiver-operating-characteristic curve (AUC) for each drug class was summarised as the mean ± standard deviation with a 95% confidence interval (Table 12).

**Table 12:** Five-fold stratified cross-validation AUC for each drug class (mean ± standard deviation and 95% confidence interval across folds).

Drug class	AUC (mean ± SD)	95% CI
Antiplatelet agents	0.952 ± 0.026	0.920–0.984
Anticoagulants	0.878 ± 0.037	0.832–0.924
Defibrinogenating agents	0.922 ± 0.015	0.904–0.940
Vasodilators	0.665 ± 0.020	0.640–0.690
Neuroprotective agents	0.753 ± 0.018	0.730–0.776
Statins	0.903 ± 0.010	0.890–0.915
Volume expanders	0.647 ± 0.011	0.634–0.660
Anti-infective agents	0.872 ± 0.015	0.854–0.891

## 3 Results

### 3.1 Baseline characteristics and drug utilisation

A total of 7,335 patients were included (mean age 62.91 ± 11.00 years; male 64.02%). The most prevalent comorbidities were hypertension (72.20%), cerebral artery stenosis (67.06%), and carotid plaque (59.60%). Atrial fibrillation was present in 2.54%, and coronary heart disease in 20.52%.

Drug utilisation was highly imbalanced: high-frequency classes included antiplatelet (98.9%) and neuroprotective (97.5%) agents; low-frequency classes included anticoagulant (5.0%), defibrinogenating (8.1%), and statin (12.1%) agents. This imbalance justified the use of class-robust algorithms and SA-based optimisation. As a consequence, overall metrics may mask weaker performance on the minority classes, which motivated the per-class evaluation and per-class threshold selection reported below.

### 3.2 Model performance comparison

To evaluate the performance of the proposed multi-label recommendation model, we compared the predicted label sets generated by the BR-SA-LightGBM model with those obtained from four widely used multi-label classification algorithms: BR-LightGBM, ML-KNN, Power-Set, MLARAM, and BRKNN. All comparative multi-label classification algorithms were implemented with default parameters. In addition, the most directly comparable baseline, BR-LightGBM, shares the same features, base learner, and per-class F1-optimal thresholds and differs only in the simulated-annealing optimisation; this matched comparison isolates the contribution of the proposed tuning and complements the classical ML-KNN, Power-Set, MLARAM, and BRKNN baselines (Table 11).

The classification accuracy and F1-score of the comparative algorithms, namely ML-KNN, Power-Set, MLARAM, and BRKNN, were no higher than 0.90. The BR-LightGBM model achieved an accuracy of 0.88, a recall of 0.87, an F1-score of 0.86, and a Hamming loss of 0.11, outperforming the other four algorithms and enabling reasonable prediction of the true label sets. In contrast, the BR-SA-LightGBM model optimised by the simulated annealing algorithm achieved an accuracy of 0.90, a recall of 0.91, an F1-score of 0.87, and a Hamming loss of 0.09. All performance metrics were superior to those of BR-LightGBM. Both BR-LightGBM and BR-SA-LightGBM were evaluated using the same per-class F1-optimal thresholds so that the comparison isolates the effect of simulated-annealing parameter optimisation. Based on all evaluation indicators, the BR-SA-LightGBM model presents significant advantages for multi-label prediction of medication regimens in patients with ischemic stroke.

Because overall metrics can mask pronounced heterogeneity between labels, per-class performance was examined (Table 9). At the fixed threshold of 0.5, the high-prevalence antiplatelet and statin labels were over-called, whereas several minority classes were markedly under-detected. After per-class threshold calibration using training-fold out-of-fold predictions, the sensitivity of these under-detected classes improved substantially (defibrinogenating agents 0.847, anticoagulants 0.622, neuroprotective agents 0.492, anti-infective agents 0.634) while high specificity was retained (Table 9); the antiplatelet and statin trade-offs were correspondingly balanced at sensitivity 0.787 and 0.741 with specificity 0.960 and 0.900; the corresponding category-specific confusion matrices are shown in Table 10. These results demonstrate that a single overall F1-score is insufficient for evaluating multi-label models under class imbalance and that class-specific operating thresholds are essential.

The five-fold cross-validation produced stable per-label AUC estimates (mean across folds: antiplatelet agents 0.952, defibrinogenating agents 0.922, statins 0.903, anticoagulants 0.878, anti-infective agents 0.872, neuroprotective agents 0.753, vasodilators 0.665, volume expanders 0.647), with narrow 95% confidence intervals (Table 12), confirming the robustness of the single-split results. The held-out ROC curves for the eight drug labels are presented in Figure 5.

**Figure 5:**
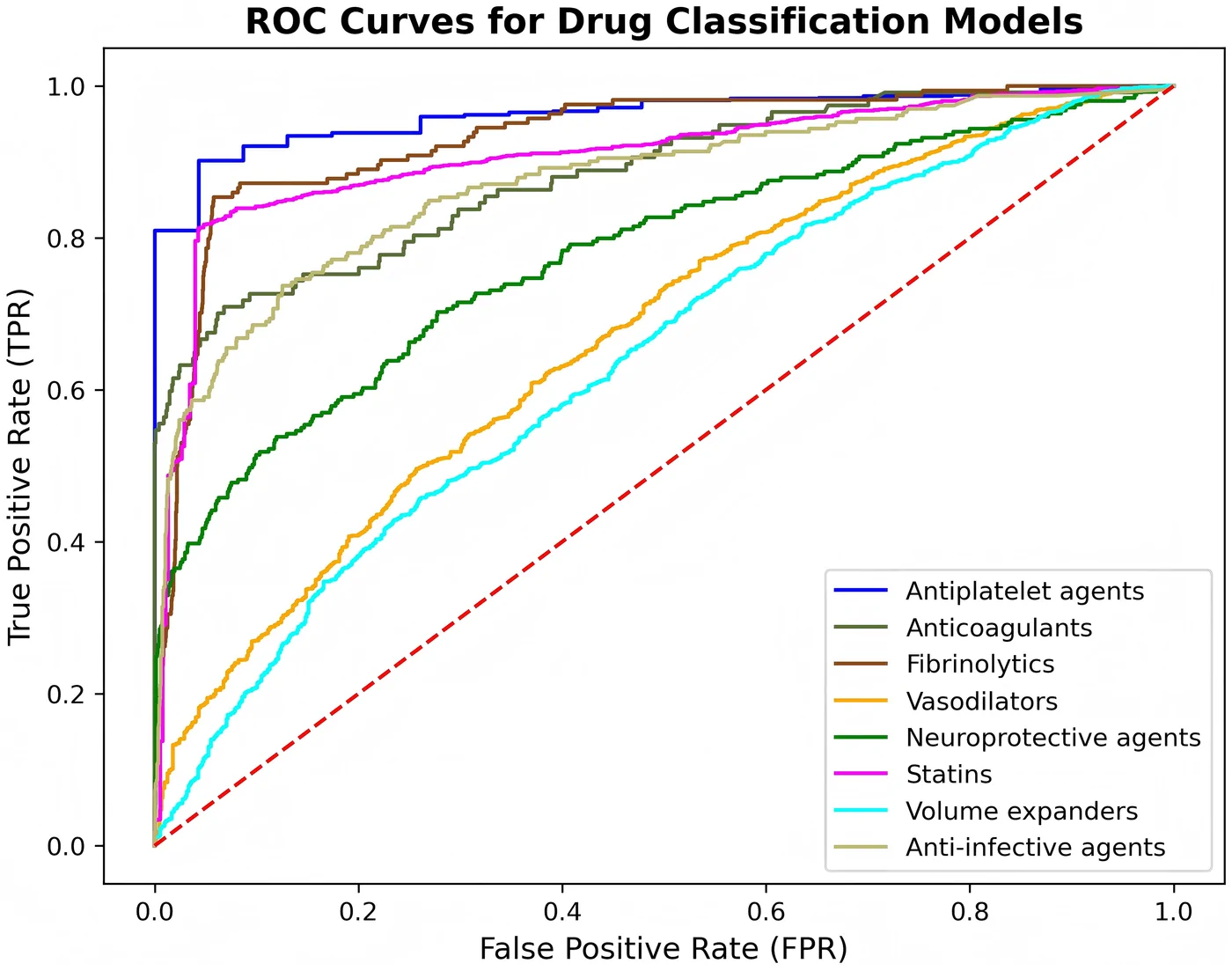
ROC diagram of drug label prediction. The horizontal axis is false-positive rate and the vertical axis is true-positive rate.

To further evaluate the predictive performance for each drug category, the ROC curves of the BR-SA-LightGBM model for individual labels in the test set are presented in Figure 5. As shown, the proposed model achieves favourable predictive performance for all drug labels.

### 3.3 Baseline characteristics and drug utilisation

To understand the relationship between the prediction of each category of drugs and patient characteristics, the feature importance of each binary classifier used to predict drug labels in the BR-SA-LightGBM model was output, and the top 5 most important features were selected to analyze the relevant variables of each category of ischemic stroke therapeutic drugs. These importance values quantify the predictive contribution of each variable within the trained model and should be interpreted as predictive associations rather than causal effects. The specific results are shown in Figure 6.

**Figure 6:**
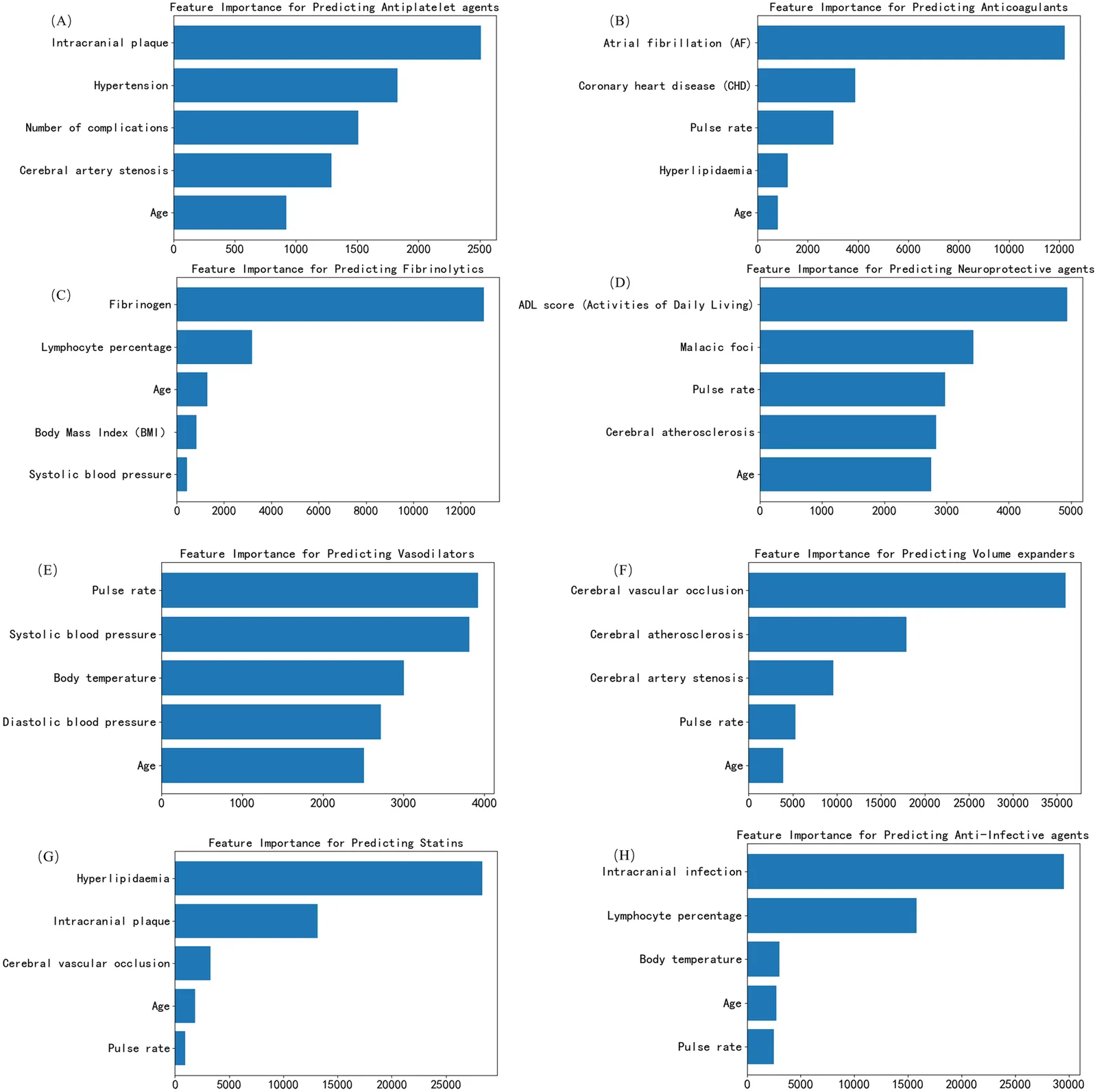
Feature importance of the top 5 ranked features is plotted for each binary classifier in the BR-SA-LightGBM model. **(A)** Feature importance for predicting antiplatelet agents. **(B)** Feature importance for predicting anticoagulants. **(C)** Feature importance for predicting fibrinolytics. **(D)** Feature importance for predicting neuroprotective agents. **(E)** Feature importance for predicting vasodilators. **(F)** Feature importance for predicting circulation-improving drugs. **(G)** Feature importance for predicting statins. **(H)** Feature importance for predicting anti-infective agents.

## 4 Discussion

### 4.1 Model performance advantages

The BR-SA-LightGBM model proposed in this study performed excellently in the multi-label recommendation task of ischemic stroke drugs, with all performance indicators superior to the comparison algorithms. The reasons are analysed as follows: (1) The BR method decomposes the multi-label problem into binary classification subproblems, reducing problem complexity and making it suitable for processing the high-dimensional characteristics of clinical data. (2) LightGBM improves training efficiency and anti-overfitting ability through GOSS and EFB technologies and can effectively mine the complex nonlinear relationship between clinical features and medication (26). (3) The SA algorithm optimizes the parameters of each base classifier, avoiding model performance limitations caused by inappropriate parameter settings and further improving prediction accuracy (27).

Compared with the BR-LightGBM model, the accuracy of BR-SA-LightGBM increased from 0.88 to 0.90, recall increased from 0.87 to 0.91, and Hamming loss decreased from 0.11 to 0.09, verifying the effectiveness of the SA algorithm parameter optimisation. The Power-Set algorithm performed poorly (accuracy = 0.64, Hamming loss = 0.35), mainly because this method regards multi-label combinations as a single category, leading to an explosion of category numbers and an unbalanced sample distribution, making it difficult to train an effective model (28). ML-KNN and BRKNN algorithms rely on the distance metric between samples and are difficult to accurately capture similarity in high-dimensional clinical data, thus limiting their performance (29).

### 4.2 Analysis of factors influencing ischemic stroke therapeutic medication

In the traditional diagnosis and treatment process, ischemic stroke patients often need to use multiple drugs for treatment (30). Physicians need to formulate reasonable medication regimens, including drug types and dosages, for each patient based on personal experience combined with clinical guidelines, monitoring indicators, and patient medical records (31). Meanwhile, physicians can provide an important basis for diagnostic medication by analysing the correlation between features and each label according to the important features corresponding to each category of ischemic stroke therapeutic drugs. To further verify the prediction performance of the BR-SA-LightGBM model for each category of drugs, we plotted the ROC curves of each label in the test set (Figure 5). This curve takes the false positive rate (FP) as the horizontal axis and the true positive rate (TP) as the vertical axis, intuitively reflecting the model's ability to distinguish each drug label. It can be seen from the figure that the ROC curves of the eight drug labels lie close to the upper left corner of the coordinate, indicating that the algorithm has good overall prediction performance; in particular, the curves for antiplatelet (cross-validated AUC 0.952), defibrinogenating (0.922), statins (0.903), anticoagulant (0.878), and anti-infective agents (0.872) lie close to the top-left corner, indicating high predictive accuracy for these classes. The curves for vasodilators (0.665) and volume expanders (0.647) deviate farther from the top-left corner, indicating relatively weaker discriminative ability for these two classes, which is consistent with the per-class results in Tables 9, 12. This was mainly because vasodilators are primarily used as adjuvant therapy with low clinical specificity (18), which is consistent with the basic principles of pharmacodynamics. The differences in predictive performance of the model were quantified by the curve morphology.

The feature importance analysis in this study shows that age, hypertension, atrial fibrillation, carotid plaque, the number of complications, and coronary heart disease are the key factors affecting the selection of various drugs for ischemic stroke, which is basically consistent with the results of many domestic and foreign studies. To more clearly present the degree of association between each drug category and core features, we output the top 5 features in the feature importance ranking of each binary classifier in the BR-SA-LightGBM model and plotted feature importance graphs (Figure 6). This presentation method refers to the visualisation logic of Lasso feature selection in the correlation analysis of type 2 diabetes datasets, taking the vertical axis as the feature importance value and the horizontal axis as different features to intuitively distinguish the core influencing factors of each category of drugs. Importantly, these features denote predictive associations rather than causal clinical determinants.

This study also examined the association patterns between drugs and symptoms, offering a valuable reference to support clinicians' medication decision-making. Overall, factors such as BMI, age, atrial fibrillation, the number of complications, coronary heart disease, and fibrinogen exert distinct influences on the selection of specific drug classes, aligning with findings from prior studies (32). Because the pathogenesis of ischemic stroke is complex with many risk factors, the selection of drug therapy regimens is targeted treatment according to risk factors (33), which conforms to the principle of precision and personalised drug therapy for ischemic stroke.

In the exploration of features influencing medication types in patients with ischemic stroke, Figure 6 demonstrates that age plays a critical role in predicting the use of all drug categories. This confirms that clinicians must prioritise age when prescribing medications: on the one hand, to assess whether a drug is suitable for the patient population; on the other hand, dosage adjustment is essential during actual administration. As shown in Figure 6A, key factors influencing antiplatelet medication use include plaque, hypertension, the number of complications, cerebral vascular stenosis, and age. These results indicate that complications such as hypertension are primary clinical considerations for antiplatelet therapy. Moreover, antiplatelet agents are the first-line strategy in patients with imaging evidence of plaque (34). Given these factors, combined antiplatelet therapy significantly improves prognosis and reduces recurrence. Figure 6B shows that complications, including atrial fibrillation and coronary heart disease, are important predictors of anticoagulant use. Coronary heart disease may induce atrial fibrillation, which increases the risk of stroke, and anticoagulants are essential for preventing recurrent stroke in patients with atrial fibrillation. Thus, anticoagulant administration reduces the incidence of ischemic stroke, consistent with clinical practice (35). Figure 6C reveals that fibrinogen is an important predictor of defibrinogenating agent use. Defibrinogenating drugs primarily reduce plasma fibrinogen levels, thereby preventing thrombus formation and progression. In ischaemic stroke patients with high fibrinogen, such agents improve cerebral hemodynamics, the coagulation-fibrinolysis system, the inflammatory response, and activities of daily living (36). Figure 6D illustrates that pulse, systolic blood pressure, and diastolic blood pressure are key features for predicting vasodilator use. Clinicians commonly evaluate vascular status based on blood pressure to guide vasodilator administration. Although vasodilators are widely used, they should be prescribed cautiously, as excessive vasodilation may paradoxically worsen ischemia (37). Figure 6E presents important features for neuroprotective drug prediction (38). Neuroprotective agents reduce brain tissue injury and improve cerebral metabolism, contributing significantly to neurological recovery. In particular, in patients with atherosclerosis and softened foci, neuroprotective drugs exert substantial effects on neural regeneration (24). Figure 6F displays key features for predicting volume-expanding agents. These drugs regulate cerebral microcirculation and blood flow, alleviate vascular occlusion and arteriosclerosis, improve cerebral stenosis, and enhance neurological function in progressive stroke, supporting their clinical application (39). Figure 6( identifies major predictors of statin use. Statins exert lipid-lowering effects and play a vital role in cardiovascular and cerebrovascular protection (40). They effectively target dyslipidemia and plaque, providing substantial benefits in acute ischemic stroke (41). Figure 6H demonstrates that infection is the dominant feature for predicting anti-infective medication. Ischemic stroke patients frequently present with concurrent infections, especially intracranial infections, which may worsen outcomes. Therefore, anti-infective agents are routinely administered in infected patients to promote recovery (42). Based on feature importance across all drug categories, machine learning-based prediction of medication selection in ischaemic stroke patients is clinically reasonable and valid. The model established in this study can serve as a reliable auxiliary decision-support tool for clinical medication recommendations.

It can also be found from the comparative analysis of Figure 6 that ischemic stroke drugs have high interdependence; the influencing factors among drugs are not completely independent, and several drugs may act on one or more risk factors, proving that there are certain constraints or dependencies among multiple drugs. For example, carotid plaque is a core feature of both antiplatelet and statin drugs, suggesting that these two drugs are often used in combination in clinical practice to exert synergistic therapeutic effects against atherosclerosis, a common risk factor. This is consistent with the overlapping analysis logic of core features of different hypoglycemic drugs in the type 2 diabetes dataset, revealing the intrinsic correlation among drugs through visual comparison of feature importance. Compared with previous studies, these important factors can be explained and analysed through pharmacological principles, which is in line with the drug use principles specified in the Chinese guidelines for the diagnosis and treatment of ischemic stroke (20).

Therefore, the BR-SA-LightGBM algorithm can make full use of existing medical data, transform the information into an auxiliary basis for physician decision-making, thereby improving physicians' work efficiency, saving national medical resources, reducing medical loss and costs, and promoting the development of auxiliary decision-making for ischemic stroke treatment.

### 4.3 Research limitations and future prospects

This study has several limitations that should be considered when interpreting its findings.

First, the cohort was derived entirely from the electronic medical record (EMR) system of a single tertiary hospital (Cangzhou Central Hospital). Although this design ensured consistent data capture, it may introduce selection bias and local overfitting to institution-specific prescribing protocols; consequently, the transportability of the model to other hospitals, regions, and levels of care remains unconfirmed. Future work will therefore conduct multicenter validation across geographically and institutionally diverse hospitals, together with temporal validation, to quantify performance drift and recalibrate the model before deployment, in accordance with contemporary reporting and risk-of-bias guidance for clinical prediction models (43, 44) and established external-validation principles (45).

Second, the model addresses only drug-class selection and does not incorporate information on drug-drug interactions (DDIs), contraindications, or adverse-event signals; recommendations issued without independent safety review may therefore carry a risk of inappropriate prescribing. In future studies, drug-interaction knowledge will be integrated into the modeling framework, and the system will be implemented as a human-in-the-loop system in which all suggestions are reviewed by clinicians, cross-checked against current guidelines, and prospectively evaluated for safety before clinical integration (46).

Third, the model does not provide dosage recommendations. Combining the present framework with dosage-related data would enable integrated recommendation of both drug type and dose, which is a prerequisite for clinically actionable decision support.

Fourth, the clinical meaning of the predicted labels should be clarified. Because the model was trained on observed physician orders extracted from the EMR, it learns the existing prescribing behaviour at the study site rather than guideline-optimal treatment. It should therefore be regarded as an auxiliary decision-support tool, not an autonomous prescribing recommendation system. Its outputs may differ from evidence-based standards, particularly where local practice departs from guidelines; formal evaluation of guideline concordance is therefore an important direction for future work.

Beyond these targeted improvements, several broader directions warrant investigation. Deep-learning approaches may be introduced to model the complex correlations between patient features and medications (47) while improving model interpretability; a dynamic recommendation model that incorporates evolving disease data during treatment could support real-time medication adjustment; and ultimately, a clinically applicable decision-support system should be developed and embedded within EMR systems to facilitate routine clinical use.

## 5 Conclusion

This study constructed a multi-label auxiliary decision-making model for ischemic stroke drug therapy based on BR-SA-LightGBM. The model addresses multi-label classification via the BR strategy, establishes base classifiers using LightGBM, and refines model parameters with the SA algorithm, thereby enabling accurate recommendation of eight therapeutic drug classes for ischemic stroke. As an auxiliary decision-support tool that learns from observed prescribing practice, it is intended to support clinical judgement. The model has demonstrated outstanding performance on large-scale clinical data, with all evaluation metrics consistently outperforming mainstream multi-label algorithms. Feature importance analysis identifies the predictive associations associated with each therapeutic drug class, offering a scientific basis to inform clinical pharmacotherapeutic decision-making. This study provides a new technical method for precision pharmacotherapy of ischemic stroke, contributing to the enhancement of diagnostic accuracy and therapeutic decision-making, has the potential to reduce medication errors, and facilitates the advancement of intelligent healthcare in the field of stroke.

## Data Availability

The original contributions presented in the study are included in the article/Supplementary Material, further inquiries can be directed to the corresponding author/s.
